# Lymph node dissection for upper tract urothelial carcinoma: A systematic review

**DOI:** 10.1080/2090598X.2020.1791563

**Published:** 2020-07-27

**Authors:** Vinson Wai-Shun Chan, Chris Ho Ming Wong, Yuhong Yuan, Jeremy Yuen-Chun Teoh

**Affiliations:** a School of Medicine, Faculty of Medicine and Health, University of Leeds, Leeds, UK; b Department of Surgery, Prince of Wales Hospital, Hong Kong, China; c Department of Medicine, McMaster University, Hamilton, ON, Canada; d S.H. Ho Urology Centre, Department of Surgery, Prince of Wales Hospital, the Chinese University of Hong Kong, Hong Kong, China

**Keywords:** Urothelial carcinoma, lymph node dissection, nephroureterectomy, UTUC

## Abstract

**Objective:**

To perform a systematic review, according to the Preferred Reporting Items for Systematic Reviews and Meta-Analyses (PRISMA) statement, investigating the role of lymph node dissection (LND) during nephroureterectomy (NU) for upper tract urothelial carcinoma (UTUC); focussing on survival and complication outcomes.

**Methods:**

A comprehensive systematic search was completed using a combination of Medical Subject Headings terms and keywords related to UTUC and LND on multiple databases. Meta-analyses were performed when outcomes were reported under the same definition in two or more studies. Where meta-analysis was not possible, outcomes were reviewed in a narrative manner.

**Results:**

A total of 21 studies were included in the qualitative analysis and 11 cohort studies in the quantitative analysis. Our review did not detect significant improvement in recurrence-free survival (RFS) (hazard ratio [HR] 0.89, 95% confidence interval [CI] 0.41–1.92), cancer-specific survival (CSS) (HR 0.89, 95% CI 0.54–1.46) and overall survival (OS) (HR 1.10, 95% CI 0.93–1.30). However, when focussing on studies only including patients with pT2/pT3 UTUC, not performing LND significantly worsened RFS (HR 2.83, 95% CI 1.72–4.66). Reports of removing more than eight lymph nodes may also provide prognostic benefits in pN0 patients. The performance of LND was not associated with a higher rate of postoperative complications (risk ratio 1.06, 95% CI 1.00–1.13).

**Conclusion:**

Overall, LND did not provide additional benefit in RFS, CSS and OS. However, there was a potential benefit in RFS in patients with muscle-invasive and advanced UTUC. LND was also not associated with increased risks of postoperative complications.

**Abbreviations:** CIS: carcinoma *in situ*; CSS: cancer-specific survival; HR: hazard ratio; LND: lymph node dissection; NU: nephroureterectomy; OS: overall survival; PRISMA: Preferred Reporting Items for Systematic Reviews and Meta-Analyses; RFS: recurrence-free survival; RoB, risk of bias; RR: risk ratio; (UT)UC: (upper tract) urothelial carcinoma

## Introduction

Urothelial carcinoma (UC) is the fifth most common tumour worldwide, yet only a limited number of them are found in the upper tract, accounting for 5–10% of cases [1]. Upper tract UC (UTUC) is often diagnosed late with more than half being muscle-invasive disease upon initial presentation. Prognosis is also worse than the lower tract counterpart, with a 5-year survival rate of <50% in advanced disease [2].

Radical nephroureterectomy (NU), either open or laparoscopic, is the mainstay of treatment for non-metastatic UTUC [3,4]. Lymph node dissection (LND) can be performed in patients with suspected regional lymph node metastasis for staging purposes. A recent study investigating the trends of LND amongst UTUC patients concluded that LND is performed more regularly during open NU, these patients are also more likely to receive adequate concomitant LND when compared to those undergoing laparoscopic NU [5]. Furthermore, whether routine LND in conjunction with NU for UTUC confers any survival benefit is unknown [3]. We decided to systemically review the evidence of LND with NU, to determine if there is any possible survival benefit of LND for patients with UTUC.

## Methods

We systematically reviewed the literature on UTUC and the role of LND in patient survival. The systematic review was performed according to the Preferred Reporting Items for Systematic Reviews and Meta-Analyses (PRISMA) statement [6].

### Literature search

A comprehensive literature search was performed using a combination of keywords (Medical Subject Headings terms and free-text words) related to ‘upper tract urothelial carcinoma’, ‘nephroureterectomy’ and ‘lymph node dissection’ up to 13 February 2020 on the Medical Literature Analysis and Retrieval System Online (MEDLINE), Excerpta Medica dataBASE (EMBASE), Cochrane Central Register of Controlled Trials database (CENTRAL), and Cochrane Database of Systematic Reviews. The search strategy is presented in Appendix 1. Additional articles were sought from the reference lists of the included studies.

### Selection criteria

All articles identified in the literature searched were screened independently by two reviewers (V.W.S.C and C.H.M.W). Conflicts were settled by a third senior author (J.Y.C.T). All cohort studies that compared radical NU with or without LND were included. Same cohorts that reported more than once were treated as one cohort and results were taken from the most recent publication. Studies of children, case reports, case series, commentaries, editorials, letter to editors, reviews, and non-English publications were excluded.

### Data collection

A piloted, standardised data entry form was devised to collect study information and data from eligible studies. Study data such as publication information, study design, inclusion and exclusion criteria, sample size, patient characteristics, and confounders, were recorded. Study results, such as complications and oncological outcomes, were also recorded. Data were collected independently by two reviewers (V.W.S.C and C.H.M.W).

### Data synthesis and statistical analysis

The primary outcome of this review was oncological survival in patients undergoing LND during radical NU for UTUC. We also compared the rate of complications between those undergoing and not undergoing LND. For these outcomes, data were analysed and pooled where there were two or more studies reporting the same outcome. The Mantel–Haenszel method was used along with the random effects model for dichotomous data, while generic inverse variance method and random effects model was used to pool time-to-event data such as hazard ratios (HRs) for survival outcomes. The results were presented as risk ratios (RRs) or HRs where appropriate, along with a Forest plot, 95% CIs and weightings. The *I*^2^ and chi-square values were utilised to detect heterogeneity between studies included for meta-analysis. Substantial heterogeneity is defined as an *I*^2^ value of >50% or a chi-square *P* < 0.10. Qualitative data were also presented in a narrative manner. Risk of bias (RoB) in these studies was assessed by the Cochrane Risk of Bias Assessment (RoB 1.0), modified to assess confounding effects of non-randomised studies, an approach recommended by the European Association of Urology (EAU) [7,8].

## Results

**Figure 1.**
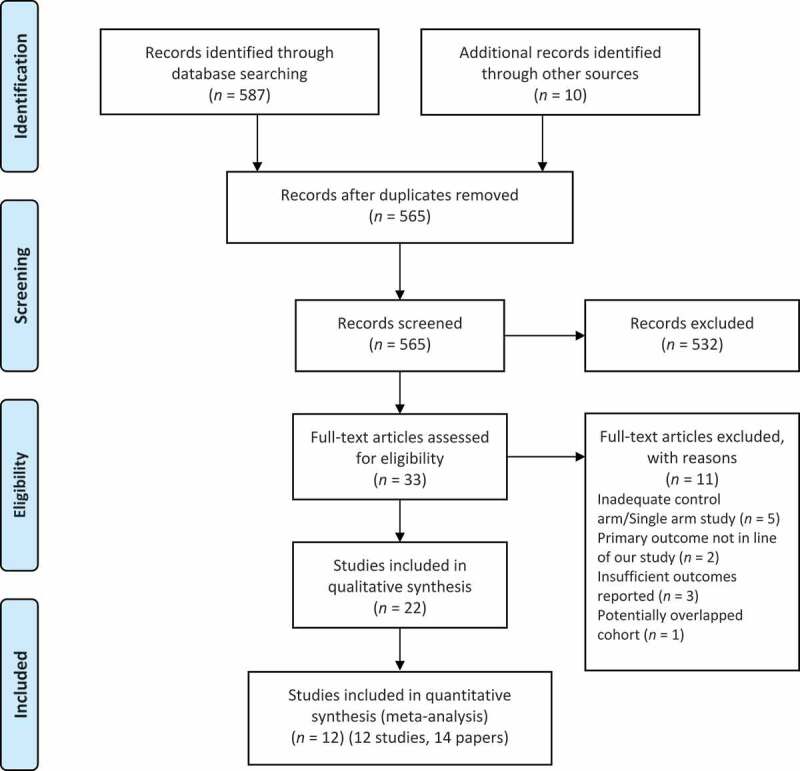
PRISMA flow diagram

**Figure 2.**
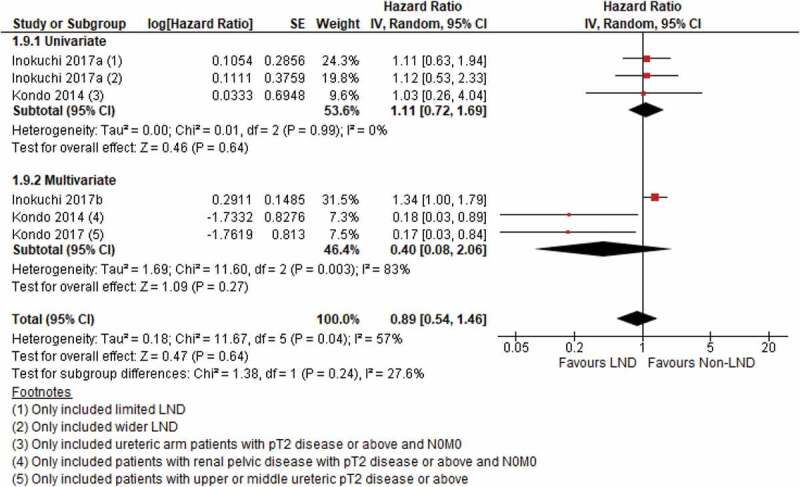
(a) ROBs of individual studies, and (b) A summary of RoBs

The PRISMA flow diagram is presented in Figure 1. A total of 587 records were identified by the literature search, and 10 additional records were sought from reference lists of the included studies. After the removal of duplicates, 565 records remained. Amongst these records, 22 were included in the qualitative synthesis, and 12 cohort studies in 14 records were included in the quantitative analysis. These studies are reported in Table 1 [5,9–21]. Five studies reported a description of their associated LND templates and these are presented in Table 2 [9,14–19]. The RoB assessments for these studies are presented in Figure 2. Owing to the lack of randomised control trials in the area, selection bias was high amongst all studies. Performance bias is unlikely in these studies. Blinding and outcome data were not well described amongst studies; hence risk of detection and attrition bias was unclear. Reporting bias was low amongst studies, while confounders like age, grade and T-stage were well accounted for in most studies. Carcinoma *in situ* (CIS) and adjuvant therapies were however less accounted for as confounders.

**Table 1.** Characteristics of included studies

Study	Year	Comparison	Country	Study type	Eligibility criteria	Total patients, *n*	LND arm, *n*	Non-LND arm, *n*	Follow-up time, months, median	Use of standardised LND template
Azawi et al. [9]	2017	Robotic/lap RNU + LND vs Robotic/lap RNU – LND	Denmark	Retro.	Upper urinary TCC of clinical stage N0M0 who underwent laparoscopic or robotic NU preoperative cN0 with suspicious LN visualised during surgery (for eLND)	277	46	231	43.5	Yes
Cho et al. [10]	2009	RNU ± LND	South Korea	Retro.	Muscle-invasive upper urinary tract TCC who underwent open NU. No distant metastasis. No unresectable lesions. No concomitant invasive bladder cancer. No LN involvement suspected on preoperative imaging studies or operative findings	152	89	63	53	No
Ikeda et al. [11]	2017	RNU ± LND	Japan	Retro.	UTUC who underwent RNU with excision of the bladder cuff. Not received neoadjuvant chemotherapy	399	222	177	43	No
Inokuchi et al. [12]	2017a	RNU ± limited/wider LND	Japan	Retro.	Clinically node negative primary UTUC who underwent RNU. No metastasis. No simultaneous bladder cancer. Complete TNM staging. No neoadjuvant chemotherapy. LN biopsy only.	823	197	626	59.8	No
Inokuchi et al. [13]	2017b	RNU ± LND	Japan	Retro.	Unilateral non-metastatic UTUC who underwent RNU. No synchronous muscle invasive bladder cancer. No history of cystectomy or urinary diversion.	2037	1046	844	45.8	No
Kanno et al. [14]	2017	RPLND vs no RPLND	Japan	Retro. matched	Renal pelvis or upper/middle ureter tumours. Renal pelvic, upper ureteric or middle ureteric tumours. No lower ureteric tumours.	64	32	32	NR	Yes
Kondo et al., [15] Kondo et al. [16] Kondo et al. [17]^a^	2007 2010 2017	RNU ± incomplete/complete LND	Japan	Retro.	Non-metastatic UTUC. Not received neoadjuvant chemotherapy.	126	50	76	NR	Yes
Kondo et al. [18]	2014	Open/lap NU ± LND	Japan	Prospect.	All patients irrespective of preoperative staging at the time of radical NU for UTUC.	166	77	89	NR	Yes
Miyake et al. [19]	1998	NU with bladder cuff resection for primary TCC ± LND	Japan	Retro.	NU with bladder cuff resection for UTUC.	72	43	35	49	Yes
Moschini et al. [5]	2017	RNU ± LND	Multinational	Retro.	Non-metastatic UTUC. Patients with complete clinical data.	1512	545	967	48	No
Pearce et al. [20]	2015	RNU ± LND	USA	Retro.	Primary diagnosis of renal pelvic or ureteric neoplasm. Adults and age available. No metastatic disease.	16,619	14,059	2,560	NR	No
Yoo et al. [21]	2017	RNU ± LND	South Korea	Retro.	UTUC who underwent RNU. No suspicion of distant metastasis and LN metastasis on preoperative imaging.	418	132	186	69 (mean)	No

eLND: extended LND; lap: laparoscopic; Prospect.: prospective; RNU: radical NU; Retro.: retrospective; RPLND, retroperitoneal pelvic LND; NR: Not reported.

^a^Same study with more than one report, the latest report was presented.

**Table 2.** Description of LND templates in the included studies

Study	Use of LND template
Azawi et al. [9]	*Left side*: left renal hilar to longitudinal midline of aorta *Right side*: right renal hilar to longitudinal midline of aorta *Caudal border*: level of the aortic bifurcation
Kanno et al. [14]	*Left side*: renal hilar and para-aortic LN *Right side*: renal hilar, paracaval, retrocaval, and intra-aortocaval LN *Cranial border*: 1–2 cm higher than the renal hilum *Caudal border*: level of the aortic bifurcation
Kondo et al., [15] Kondo et al. [16] Kondo et al. [17]^a^	*Right*: right renal hilar, paracaval, retrocaval, inter-aortocaval *Left*: left renal caval, para-aorta, aortic bifurcation
Kondo et al. [18]	Renal pelvis: *Left*: left renal hilar, para-aorta down to the level of IMA *Right*: right renal hilar, para-caval, Interaortocaval down to the level of IMA Upper 2/3 ureter: *Left*: left renal hilar, para-aorta down to the level of aortic bifurcation *Right*: right renal hilar, para-caval, Interaortocaval down to the level of aortic bifurcation Lower 1/3 ureter: Ipsilateral common iliac, external iliac, internal iliac, obturator
Miyake et al. [19]	Renal pelvis or upper ureter: from para-aorta to vena cava *Cranial border*: renal hilum *Caudal border*: IMA Mid-ureter: from para-aorta to vena cava *Cranial border*: renal hilum *Caudal border*: bifurcation of the common iliac artery Lower ureter: Ipsilateral pelvic nodes on the ipsilateral side (Greater extent carried out when multiple tumours were located in different areas of the ureter)

IMA: inferior mesenteric artery.

^a^Same study with more than one report, the latest report was presented

### Recurrence rate

**Figure 3.**
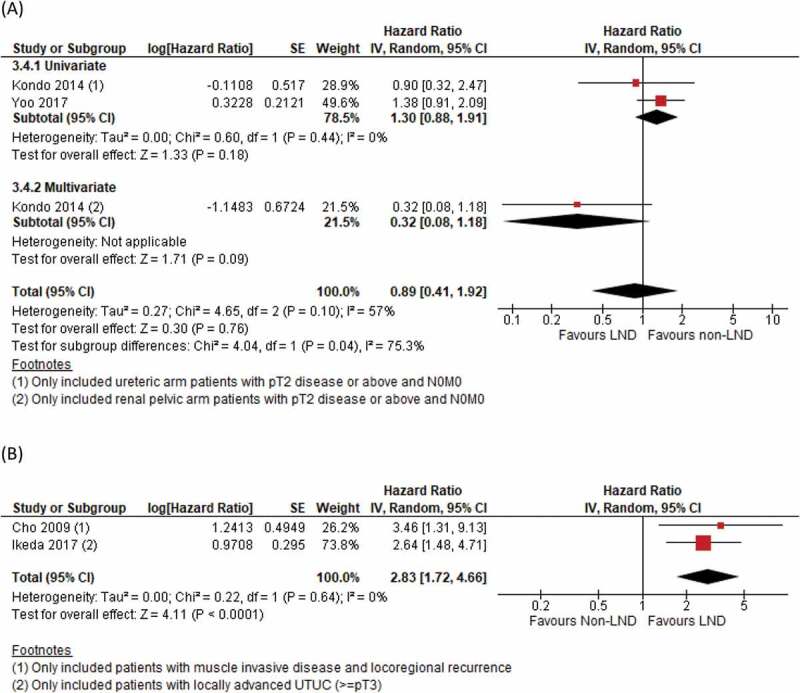
Rate of recurrences at ≥36 months

We identified three studies in total that reported the number of recurrences. At follow-up of ≥36 months our meta-analysis of 577 patients did not detect any significant reduction in the recurrence rate of patients undergoing LND (RR 1.14, 95% CI 0.83–1.57; *P* = 0.41) (Figure 3). There was no heterogeneity between the included studies.

### Recurrence-free survival (RFS)

**Figure 4.**
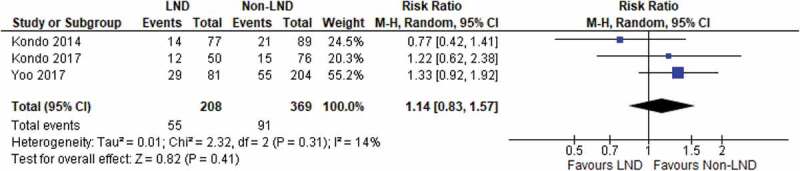
(a) RFS, non-LND as reference, (b) RFS, LND as reference

There were four studies reporting adjusted HRs for RFS. Two used a non-LND group as reference, and we did not detect any significant differences in RFS (HR 0.89, 95% CI 0.41–1.92; *P* = 0.76) (Figure 4(a)). The study by Kondo et al. [18] only included patients with ≥T2 disease, and the contrasting effect in HRs between univariate and multivariate analysis highlights the potential impact of disease status on treatment effects, contributing to the substantial heterogeneity, as evident by the subgroup analysis. In the remaining two studies focussing on muscle-invasive (pT2) and advanced (pT3) UTUC using the LND group as reference, our meta-analysis demonstrated a significantly increased risk of recurrence if LND was not performed, with no significant heterogeneity found within the included studies (HR 2.83, 95% CI 1.72–4.66; *P* < 0.001) (Figure 4(b)).

### Cancer-specific survival (CSS)

**Figure 5.**
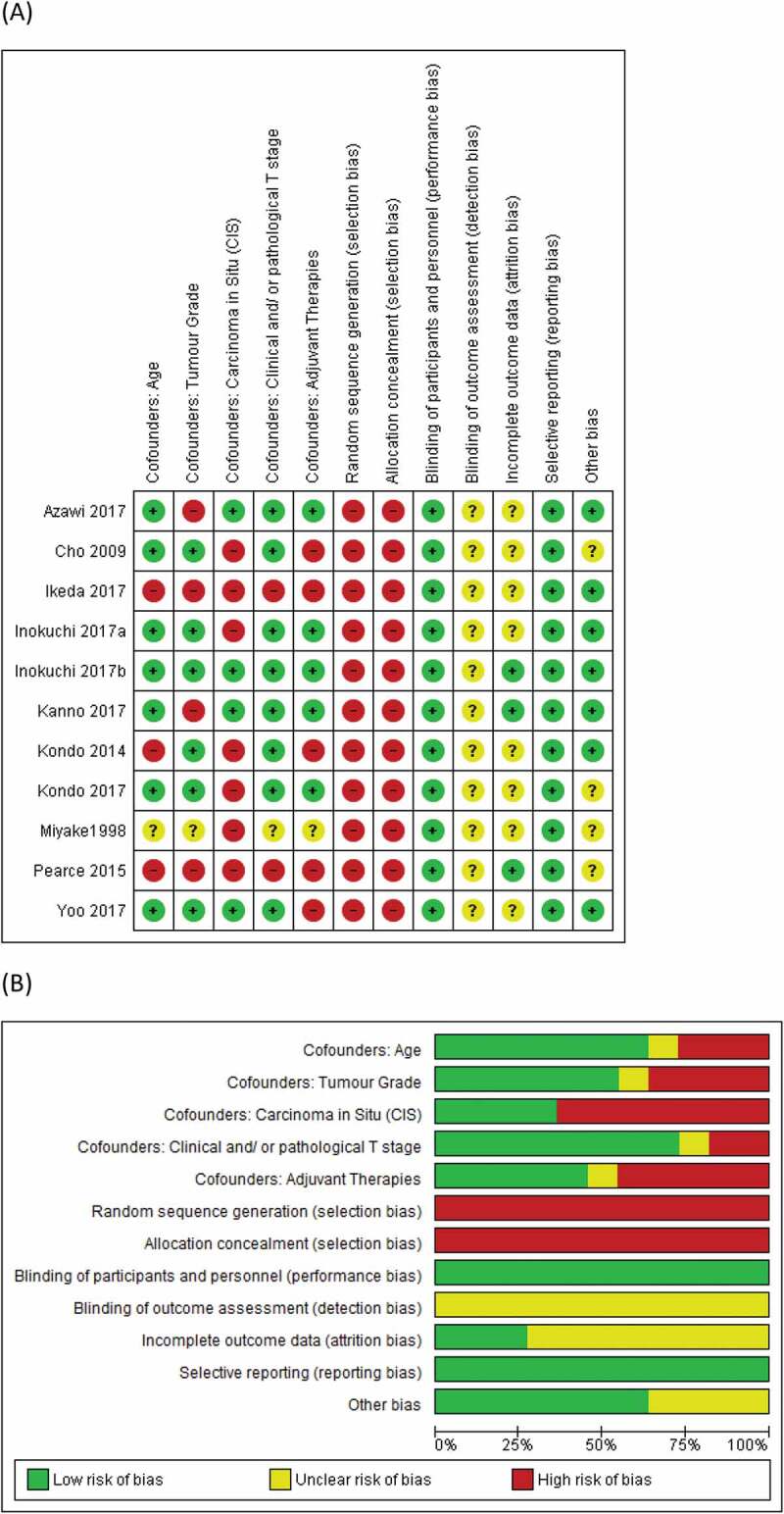
CSS

Our meta-analysis identified four studies reporting CSS in patients undergoing LND. Two individual studies on patients with ≥T2 disease showed that LND was associated with better CSS [17,18]. However, the results became insignificant after incorporating the third and fourth studies, which included all T-stages for CSS (HR 0.89, 95% CI 0.54–1.46; *P* = 0.46) (Figure 5), contributing to substantial heterogeneity between studies. Furthermore, Ikeda et al. [11] reported a significantly increased risk of cancer-specific death if LND was not performed in patients with ≥T3 disease. (HR 3.17, *P* = 0.001). Both Roscigno et al. [22] and Kondo et al. [18] reported the association between increased lymph node yield and its benefits on CSS. Roscigno et al. [22] dichotomised the number of lymph nodes removed to eight or above, the HR for CSS reduced significantly to 0.49 (*P* < 0.01) with increasing number of lymph nodes. Kondo et al. [18] reported a nearly significant protective effect with increasing number of lymph nodes removed (HR 0.92, 95% CI 0.82–1.01; *P* = 0.05) in patients with ≥pT2 renal pelvic cancer. Both studies suggested potential benefits to CSS when more lymph nodes were removed.

### Overall survival (OS)

**Figure 6.**
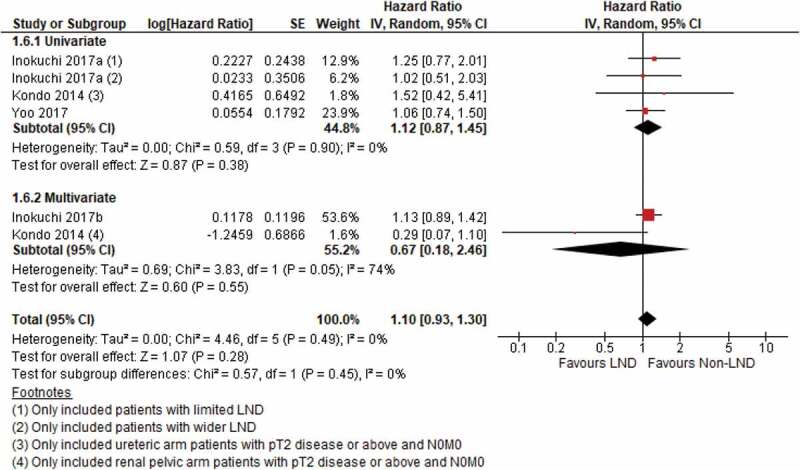
OS

There were four studies reporting OS, and no significant difference was detected between the LND and non-LND groups (HR 1.10, 95% CI 0.93–1.30) (Figure 6). No heterogeneity was found between the included studies. A further study by Miyake et al. [19] reported that the 1-, 3- and 5-year OS rates were 91%, 73%, and 58%, respectively in patients who underwent LND vs 83%, 65% and 50%, respectively in patients who did not undergo LND. However, Kondo et al. [18] found a significant improvement in OS when more lymph nodes were removed (HR 0.92, 95% CI 0.83–0.99; *P* = 0.03).

### Intraoperative characteristics

While open procedures are common, laparoscopic and robot-assisted LNDs are emerging procedures. A study by Kanno et al. [14] assessed intraoperative characteristics and found a significantly longer operative time and non-significantly lower estimated blood loss during laparoscopic LND in radical NU for upper ureteric and renal pelvic cancer. A nationwide study, by Pearce et al. [20] in the USA, reported a higher intraoperative complication rate in the LND group (4.34%) when compared to the non-LND group (3.76%); however, the results were not statistically significant.

### Postoperative complications

**Figure 7.**
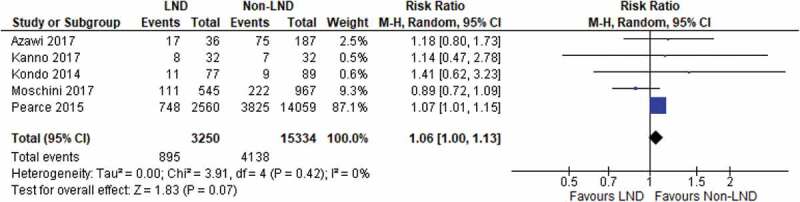
Postoperative complications

There were five studies reporting on the rate of complications during radical NU and LND. Across 18 584 patients in five studies, performing LND was not associated with higher rates of postoperative complications (RR 1.06, 95% CI 1.00–1.13; *P* = 0.07) (Figure 7). No heterogeneity was found between the included studies. Of the major complications being reported haemorrhage, gastrointestinal, cardiac, urinary and lymphatic complications were the most common. Further LND-specific complications reported by Kondo et al. [18] included numbness of the thigh, lymphorrhoea and chyle fistula, although lymphorrhoea was also observed in one patient from the non-LND group. When we included laparascopic and robotassistedLNDs only, there was also no significant differencebetween the LND and non-LND groups for postoperativecomplications [9,14].

## Discussion

LND is a common procedure performed in potentially curative cancer surgery. The lymph nodes being excised also allows proper histological assessment and this may be important for staging purposes. However, the role of LND in patients with UTUC is limited, mainly because of the lack of evidence in survival benefits.

In the present study, we performed a comprehensive literature search for LND in patients with UTUC. Although we identified 11 comparative studies, none of them were randomised controlled trials. The quality of the studies included was generally low, as reflected by the RoB assessment. As UTUC is a rare disease, to a certain extent, it is understandable why there is such a lack of high-quality evidence in this area. A proper multicentre randomised controlled trial investigating the role of LND with NU in patients with UTUC is urgently needed.

UTUC can occur anywhere from the kidney to the lower ureter, together with the laterality of UTUC, the LND template can be complicated and difficult to standardise. Upon review of the literature, we recognised large variations in the indication for LND, as well as the LND templates, across the different studies. Interestingly, Furuse et al. [23] showed that the use of a standardised and systematic template LND (compared to a non-systematic LND approach) was able to improve survival outcomes in patients with UTUC. Riscigno et al. [22] also demonstrated that a minimum yield of eight lymph nodes led to significant benefit in RFS and CSS in pN0 patients, highlighting the importance of a standardised anatomical template for LND. These results showed that a larger extent of LND might favour oncological outcomes; a lack of standardisation affects the reliability, as well as the interpretation, of the results.

In the present study, we found a potential benefit in RFS in the two studies using the LND group as reference (Figure 4(b)). When the two studies by Ikeda et al. [11] and Cho et al. [10] investigated the role of LND only in T2 and ≥T3 disease respectively, not performing LND increased the risk of recurrence. Ikeda et al. [11] concluded similar results for cancer-specific deaths. This suggests a potential role of LND in patients with more advanced disease. On the other hand, the present analysis was limited by the small number and low quality of the studies included. We also did not detect any significant benefit of LND in terms of recurrence rate, CSS and OS. Moreover, our present meta-analysis did not demonstrate an increase in complications in patients undergoing LND Figure 7[9,24]. To sum up, we believe the current evidence does not justify the indication of routine LND in patients with UTUC.

The recent POUT trial [25] (ClinicalTrials.gov, NCT01993979) recruited 261 patients who were randomised to be under surveillance or to receive 21-day-cycles of chemotherapy after NU for UTUC. The authors were able to demonstrate a significant benefit in disease-free survival of adjuvant chemotherapy in patients with pN0 and ≥pT3 disease, but not in those with pN+ disease. In this study, patients either did not receive LND, or only received limited LND. Whether the adoption of a systematic and standardised LND could optimise the cancer control in patients with node-positive disease is unknown. This will be an interesting area that demands more high-quality studies in the future.

The present study was a comprehensive systematic review investigating the role of LND during radical NU for patients with UTUC. However, there are several limitations to our present study. First, given the rarity of UTUC, there was a lack of high-quality evidence in this area and this is well reflected by our RoB assessment. Second, there was a lack of standardisation across the studies in terms of the LND template, surgical approach, and the use of chemotherapy. This could affect the reliability and the interpretation of our present results. Third, most studies included in our present review were conducted in East Asia, with the role of LND unclear in other populations. A large-scale, prospective, multicentre randomised trial is urgently needed to investigate the role of LND in patients with UTUC. Stratification according to the laterality and location of the tumour, as well as the disease status, will be able to help us understand more about the treatment effects of LND in patients with UTUC.

## Conclusion

Our systematic review concluded that LND did not lead to a benefit in recurrence rate, CSS, or OS. We observed a potential benefit of LND on RFS in muscle-invasive and advanced UTUC; however, this was limited by the small number and low-quality of the studies. Furthermore, there was no increased risk of postoperative complications when LND was performed, compared to the non-LND group. In conclusion, we do not recommend routine LND in patients with UTUC undergoing NU.

## Appendix 1

Search Strategy

Database: OVID Medline Epub Ahead of Print, In-Process & Other Non-Indexed Citations, Ovid MEDLINE(R) Daily and Ovid MEDLINE(R) 1946 to Present, Embase <1974 to 2020 February 13>, EBM Reviews - Cochrane Central Register of Controlled Trials <January 2020>, EBM Reviews - Cochrane Database of Systematic Reviews <2005 to February 11, 2020>

Search Strategy:---------------------------

1 exp Carcinoma, Transitional Cell/ or exp transitional cell carcinoma/ (45697)

2 exp Ureteral Neoplasms/ or exp ureter tumor/ (8279)

3 (transitional cell adj5 (cancer* or carcin* or malig* or tumor* or tumour* or neoplas* or papilloma*)).tw,kw. (24645)

4 ((urothelial or urothelium) adj5 (cancer* or carcin* or malig* or tumor* or tumour* or neoplas* or papilloma*)).tw,kw. (38404)

5 ((upper urinary tract adj2 (cancer* or carcin* or malig* or tumor* or tumour* or neoplas* or papilloma*)) or UTUC).tw,kw. (4950)

6 ((renal or kidney*) adj2 (pelvis or calyces) adj5 (cancer* or carcin* or malig* or tumor* or tumour* or neoplas* or papilloma*)).tw,kw. (4464)

7 (ureter* adj5 (cancer* or carcin* or malig* or tumor* or tumour* or neoplas* or papilloma*)).tw,kw. (10422)

8 or/1-7 (81775)

9 exp Nephroureterectomy/ (5227)

10 (nephroureterectom* or nephro-ureterectom* or heminephroureterectom*).tw,kw. (8144)

11 or/9-10 (9463)

12 8 and 11 (6823)

13 exp Lymph Node Excision/ or exp lymph node dissection/ (106697)

14 (lymphadenectom* or lymphoadenectom* or LND or LNE).tw,kw. (48514)

15 (((lymph* adj3 node*) or LN) and (excision* or dissect* or resect* or extirpation* or remov*)).tw,kw. (156154)

16 exp Lymph Nodes/pa, su (47750)

17 or/13-16 (260216)

18 12 and 17 (916)

19 (child/ or Pediatrics/ or Adolescent/ or Infant/ or adolescence/ or newborn/ or (baby or babies or child or children or pediatric* or paediatric* or peadiatric* or infant* or infancy or neonat* or newborn* or new born* or adolescen* or toddler*).tw.) not (adult/ or aged/ or (aged or adult* or elder* or senior* or men or women).tw.) (4271894)

20 18 not 19 (915)

21 (exp animals/ or exp animal/ or exp nonhuman/ or exp animal experiment/ or animal model/ or animal tissue/ or non human/ or (rat or rats or mice or mouse or swine or porcine or murine or sheep or lambs or pigs or piglets or rabbit or rabbits or cat or cats or dog or dogs or cattle or bovine or monkey or monkeys or trout or marmoset$1).tw.) not (humans/ or human/ or human experiment/ or (human* or men or women or patients or subjects).tw.) (10427684)

22 20 not 21 (915)

23 limit 22 to english language [Limit not valid in CDSR; records were retained] (813)

24 remove duplicates from 23 (614)

25 ((Bladder or vesical) not (renal or kidney or ureter* or upper urinary tract or urothelial or urothelium or Transitional Cell)).ti. (163651)

26 24 not 25 (587)

***************************
